# Evaluation of a commercial web-based weight loss and weight loss maintenance program in overweight and obese adults: a randomized controlled trial

**DOI:** 10.1186/1471-2458-10-669

**Published:** 2010-11-03

**Authors:** Clare E Collins, Philip J Morgan, Pennie Jones, Kate Fletcher, Julia Martin, Elroy J Aguiar, Ashlee Lucas, Melinda Neve, Patrick McElduff, Robin Callister

**Affiliations:** 1 Nutrition and Dietetics, School of Health Sciences, Faculty of Health, The University of Newcastle, Callaghan, NSW, 2308 Australia; 2 School of Education, Faculty of Education & Arts, The University of Newcastle, Callaghan, NSW, 2308 Australia; 3 SP Health Co. Pty Ltd, North Sydney, NSW, Australia; 4 School of Biomedical Sciences and Pharmacy, Faculty of Health, The University of Newcastle, Callaghan, NSW, 2308 Australia; 5 Hunter Medical Research Institute, Faculty of Health, University of Newcastle, NSW 2308, Australia

## Abstract

**Background:**

Obesity rates in adults continue to rise and effective treatment programs with a broad reach are urgently required. This paper describes the study protocol for a web-based randomized controlled trial (RCT) of a commercially available program for overweight and obese adult males and females. The aim of this RCT was to determine and compare the efficacy of two web-based interventions for weight loss and maintenance of lost weight.

**Methods/Design:**

Overweight and obese adult males and females were stratified by gender and BMI and randomly assigned to one of three groups for 12-weeks: waitlist control, or basic or enhanced online weight-loss. Control participants were re-randomized to the two weight loss groups at the end of the 12-week period. The basic and enhanced group participants had an option to continue or repeat the 12-week program. If the weight loss goal was achieved at the end of 12, otherwise on completion of 24 weeks of weight loss, participants were re-randomized to one of two online maintenance programs (maintenance basic or maintenance enhanced), until 18 months from commencing the weight loss program. Assessments took place at baseline, three, six, and 18 months after commencing the initial weight loss intervention with control participants repeating the initial assessment after three month of waiting. The primary outcome is body mass index (BMI). Other outcomes include weight, waist circumference, blood pressure, plasma markers of cardiovascular disease risk, dietary intake, eating behaviours, physical activity and quality of life.

Both the weight loss and maintenance of lost weight programs were based on social cognitive theory with participants advised to set goals, self-monitor weight, dietary intake and physical activity levels. The enhanced weight loss and maintenance programs provided additional personalized, system-generated feedback on progress and use of the program. Details of the methodological aspects of recruitment, inclusion criteria, randomization, intervention programs, assessments and statistical analyses are described.

**Discussion:**

Importantly, this paper describes how an RCT of a currently available commercial online program in Australia addresses some of the short falls in the current literature pertaining to the efficacy of web-based weight loss programs.

Australian New Zealand Clinical Trials Registry (ANZCTR) number: ACTRN12610000197033

## Background

Obesity is a major health problem in Australia [1], just as it is internationally, and is associated with a range of adverse physiological and psychological consequences [2]. By 2000, 67% of men and 52% of women [3] in Australia over 24 years of age were measured objectively as being overweight (Body Mass Index (BMI) >25) or obese (BMI >30). The 5-year AusDiab follow-up study demonstrated that 200,000 adults progress from being overweight to obese each year [2]. Consequently, developing strategies to target obesity treatment and prevention is a national priority.

In 2008, the total financial cost of obesity in Australia was estimated to be $8.28 billion. This increases to $58.2 billion if indirect costs such as loss of wellbeing are included [4]. Although prevention of excess weight gain is the ultimate solution, even moderately effective treatments have the potential to improve health and curb heath care costs. For example, the Diabetes Prevention Program has shown that a 57% reduction in the risk of developing type-2 diabetes can be achieved by a weight loss of 5% of initial weight, if maintained for two years [5].

The proportion of the population desiring to lose weight, for either health or aesthetic reasons, is increasing [6] and has contributed to the growth of commercial weight programs, such as Weight Watchers and Jenny Craig and relatively new internet-based weight loss programs such as The Biggest Loser Club. However, industry-delivered weight loss programs, including those that are web-based, are rarely evaluated using rigorous research designs [7]. Further, health professionals may be hesitant to recommend commercial weight loss programs unless they are evidence-based and/or they have been shown to be efficacious.

Accessing health services for obesity treatment is limited with inequitable services relative to population distribution. Internet-based weight loss programs have the potential to provide a service to large numbers of people, be widely accessible (metropolitan, regional, rural, remote, international) and cost effective for the individual. Web-based weight management is under-explored relative to the rapid uptake of the Internet by adults in Australian home environments. Currently in Australia 72% of households have access to the Internet and although there are lower access rates in rural and remote areas and among households with lower weekly incomes [8], we have previously shown that a web-based weight loss program is of interest to those in 'hard-to-reach' groups such as males, younger people, living in rural and remote locations [9]. Internet programs may overcome traditional barriers to weight loss treatment due to 24 hour a day accessibility, affordability and anonymity for those who may avoid seeking treatment due to embarrassment or other reasons [10]. It can also provide a forum for social support through e-mail, bulletin boards, chat rooms, group forums and web-hosted meetings [11]. Importantly, it can minimize participant burden and irritation associated with group sessions or clinic visits [12], particularly time spent in waiting rooms, and has the potential to be used in a broad range of settings to optimise weight outcomes for patients.

## The evidence base for web-based weight loss

We recently published a systematic review and meta-analyses examining the efficacy of Internet-based weight loss programs [13]. Of the 18 studies identified, seven were assessed for effectiveness based on percentage weight change and four were deemed effective. However, it was not possible to clearly establish the efficacy or the effectiveness of web-based interventions on weight loss due to the heterogeneity of study designs. Whether Internet-based programs are effective at achieving weight loss that is subsequently maintained is also largely undetermined [13]. Although greater use of website features may increase weight loss, we do not know which specific components are more important or can reduce attrition. Therefore, examining the efficacy of commercially available Internet delivered weight management interventions, and determining the specific contributions of self-monitoring of diet, physical activity and body measurements, as well as use of features such as online support, are warranted.

## Study aim

The aim of this paper is to report the study protocol used in an RCT examining the efficacy of a commercially available online weight loss program on initial weight loss and maintenance of lost weight up to 18 months.

## Design and methods

### Ethics approval

The University of Newcastle's Human Ethics Research Committee approved this study. Written informed consent was obtained from all participants prior to their enrolment.

### Design - Randomized Control Trial

A prospective randomized controlled trial conducted in adult participants that were classified as overweight or obese. The study consisted of two phases: a weight loss phase (Phase 1) followed by a maintenance of lost weight phase (Phase 2).

#### Phase 1 - Weight-loss interventions

Participants were initially randomized to one of three groups:

(1) Group 1, wait-list control; subsequently re-randomized to Group 2 or 3 after an initial 12-week control period;

(2) Group 2 followed the standard (Basic) commercial online weight loss program;

(3) Group 3 followed an extra support (Enhanced) version of the online weight loss program and received system-generated personalized feedback, based on diary entries and website use.

At the end of the initial 12 weeks of weight loss, those in Groups 2 and 3 could choose to undertake a further 12 weeks of weight loss or if they had achieved their goal weight, which was a loss of a minimum of 10% of initial body weight, they were re-randomized to one of two maintenance of lost weight programs (Phase 2).

#### Phase 2

Maintenance Group 1 (M1) was usual care whereas Maintenance Group 2 (M2) was enhanced support. Phase 2 will run for 12 months for those commencing after 6 months of weight loss or for 15 months for those entering after only 12 weeks of weight loss.

### Outcome measures

Assessments were at baseline, 3, 6 and 18 months. The Phase 1, Group 1 control participants received an extra assessment at the end of the initial waiting list control period and were then also evaluated at 3, 6 and 18 months from the start of weight loss, meaning they will participate for a total of 21 months.

### Participants and recruitment

Participants were recruited in the Hunter region of NSW, Australia through advertising (radio, TV, newspapers, flyers in GP clinics, University website, workplace-based emails) and using the University media unit. Inclusion and exclusion criteria are summarised in Table 1.

**Table 1:** Inclusion and exclusion criteria for recruitment into the Biggest Loser Club RCT.

Inclusion Criteria	Exclusion Criteria
Aged between 18 and 60 years	Currently pregnant or trying to become pregnant
BMI between 25 and 40 kg/m^2^	History of major medical problems such as heart disease or diabetes requiring insulin treatment
Agree to not participate in other weight loss programs during the study	Orthopaedic or joint problems that are a barrier to physical activity
Pass a health-screening questionnaire and available for assessment sessions [23]	Recent weight loss of ≥ 4.5 kg in previous 6 months
Have access to a computer with e-mail and Internet facilities	Taking medications that might be affected by weight loss or affect weight loss

### Randomization

**Figure 1:**
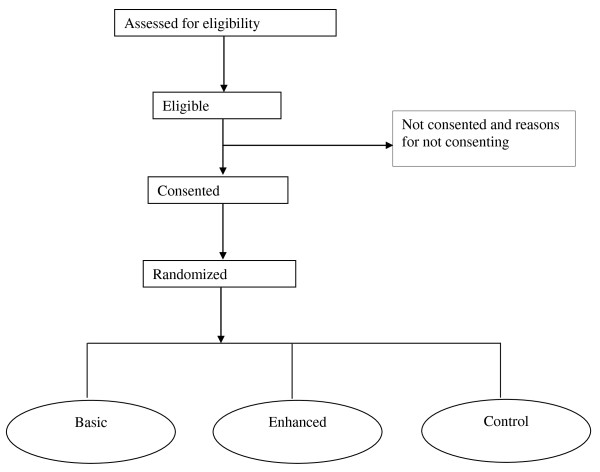
**Flow of participants into the three baseline groups of the web-based weight loss RCT**.

Once written consent was obtained and baseline assessments completed participants were randomly allocated to one of the three Phase 1 groups (Figure 1) using a stratified randomized block design. Participants were stratified by gender and category of body mass index (25 to <30; ≥30 to <35 or ≥35 to 40) using blocks of variable length (either 3 or 6). Subjects received the next envelope in the sequence based on their stratified BMI and gender group. Envelopes were prepared ahead of time, sealed and distributed by a researcher not involved in data collection. Subjects were asked to not open their allocation envelope until they returned home. Subjects were re-randomized in the same way at the end of the weight loss phase to one of the two maintenance arms.

### Sample size

Based on having 90% power to detect a significant difference in BMI between weight loss groups of 1.5 kg/m^2^, assuming the SD of BMI is 1.5 and using a two-sided significance level of 0.05, a sample size of 48 participants (24 males and 24 females) is needed for each maintenance group at 18 months. This sample size is within the range of other web-based weight loss intervention studies [13].

## SP Health Weight Management Program

SP Health Co PTY LTD is a commercial online weight loss program provider, which runs a number of Internet-based programs in Australia, UK and Asia including The Biggest Loser Online Diet Club Australia http://www.biggestloserclub.com.au

### Weight loss and weight maintenance Interventions

#### Theoretical framework for development of online features

Achieving and maintaining weight loss requires behaviour change. Bandura's Social Cognitive Theory [14] proposes that behaviour change is influenced by environmental factors, personal factors, and attributes of the behaviour itself. This interaction is referred to as 'reciprocal determinism', as each factor may affect or be affected by the others. The online program targeted key mediators including self-efficacy (e.g. goal setting, self-monitoring of weight, body measurements, exercise and diet), outcome expectations (e.g. knowledge-based web components), modelling (e.g., interactive website features and demonstrations) and social support (i.e. forums, blogs, feedback, email and/or telephone contact).

### Phase 1 - Weight-loss interventions

The interventions are delivered online for 12 weeks, with new program content distributed to the user weekly for both programs. At the end of 12 weeks participants could choose to repeat the same 12-week weight-loss program, or continue for an additional 12 weeks with the accumulated program content all available, or transfer to weight-loss maintenance if they lost a minimum of 10% of their baseline weight.

#### Group 1 - Wait-list control

The initial 12-week waitlist control group was a separate control arm. These subjects were assessed at baseline and 12 weeks, and then re-randomized to Group 2 or Group 3. The time frame for the control was limited to 3 months as it is unrealistic to expect people who volunteer for a weight-loss study in anticipation of achieving weight loss to comply with waiting for longer than 3 months. They did not have access to the study website and were also asked to refrain from undertaking any other weight-loss program during this time.

#### Group 2 - Basic online program

These participants had free access to the study website for the duration of the project. This online program has the following features:

• Individualised daily Calorie targets to facilitate 0.5-1 kg weight loss per week, based on participants weight, height and activity level

• Online food and exercise diaries and search engines to facilitate entry of food data

• Weekly Calorie-controlled, low fat menu plans and grocery list

• Weekly physical activity plan according to exercise preferences

• Weekly educational tips and challenges

• Social support via online forums

• Daily and weekly calculations of energy balance and nutrition summaries referenced to recommended nutrient targets

• Weekly email newsletters notifying the user of new content relevant to their point in the program

• Self-monitoring of reported body weight, waist and hip girths, with automated weekly reminders for entering weight

• Goal setting options and graphical display of changes in body measurement data and body (BMI) silhouette.

#### Group 3 -Enhanced online program

These participants had access to the online program described above plus received automated, computer-generated, personalized feedback based on their diet and physical activity diaries, their use or lack of use of the standard website features, and the level of success of their weight loss.

This "enhanced" online program has the following features additional to the basic program:

• A personalised enrolment report (system-generated) which suggests weight loss goals and prioritizes key behaviours the user will need to change to be successful at weight maintenance

• Weekly automated (system-generated) personalized feedback for key elements of nutrition and physical activity levels based on entries made in the online diary, including strategies to help the user change unhelpful eating behaviours identified during enrolment.

• Weekly (system-generated) personalized feedback reporting on the level of success of their weight loss journey, and general use of website features,

• Reminder schedule for compliance to the diary, site visitations and a weekly weigh-in escalating with urgency (email, text messages and personal phone calls).

### Phase 2 - Maintenance of lost weight

This phase commenced at the completion of the weight loss phase.

#### Group M1 Basic maintenance of lost weight program

• Continued access to the online program for a further 12 months, including online forum access.

• Adjusted daily Calorie target for weight maintenance.

• Weekly menu plans and exercise programs according to individual preferences, adjusted for the user's maintenance Calorie target.

• Continuation of weekly educational tips and challenges based upon food, activity and lifestyle, including four consecutive weeks of maintenance-specific content.

• A certificate to celebrate the achievement of goal weight or reaching the maintenance phase.

• Weekly email and phone text prompts to continue entering body weight and other body measurements.

• Maintenance-specific feedback on weigh-ins including warnings if the user's weight is creeping up.

• Access to an online information page detailing evidence-based behavioural strategies to enhance the likelihood of weight loss maintenance (e.g., eat breakfast; continue to monitor diet and physical activity; follow a low fat, low energy dense eating plan; be active for one hour a day; limit take-away and fast food; moderate week-end eating; react quickly when weight gain is observed).

#### Group M2 Enhanced maintenance of lost weight program

• All of the M1 program plus the following:

• Initial "welcome to weight maintenance phase" phone call from a trained consultant.

• Weekly and monthly system-generated, personalized reports summarizing energy balance and weight loss progress, website activity, and achievement of nutrition and physical activity targets.

• Regular questionnaires to help the user identify and prioritize behaviors (diet, physical activity and lifestyle-related) that can help achieve weight maintenance and prevent rebounds.

• Continuation of email, text and phone reminders to self-monitor weight, diet and physical activity, adjusted for maintenance time frame.

• Specific "relapse" weight loss program if weight rebounds by more than 3% of their baseline weight, including an initial phone call from a trained consultant.

• Congratulation emails for successful maintenance of lost weight.

## Quality Control

Monitoring the quality of the trial is essential to ensure a robust study and maintain internal validity. Several procedures were employed to optimize the quality of the study and maximize validity and reliability of the program delivery and outcome assessments. These are described below.

### Assessor blinding

Assessors of the main outcome measures are blinded to participant group allocation. These include those conducting anthropometric measurements, blood pressure and collecting blood samples. Participants are asked not to inform data collection personnel of their group allocation.

### Written documentation

All written documentation, including assessment protocols and letters sent to participants were standardised and subject to institutional ethics committee approval.

### Training

Data collection personnel involved in the assessments were trained prior to the assessments. Where possible the same assessors were used for all assessments. Consultants from SP Health were also trained prior to commencing reminder and maintenance phone calls to ensure consistency. Phone logs were recorded for quality purposes.

### Regular Teleconferences

Members of the BLC research team participated in weekly teleconferences with SP Health, the organisation delivering the online programs for the first 6 months and then fortnightly until all subjects were in the maintenance of lost weight phase. All teleconferences were minuted for reference.

### Instrument calibration

In order to ensure accurate and consistent measurements, the study weight scale was professionally calibrated once a year and the height scale checked and recalibrated daily before measurements commenced.

#### Demographic Data

Questionnaires to obtain current medical conditions and medications and were completed by participants at each data collection session. Smoking status and postcode as a proxy for socio-economic status were collected once.

#### Measurements

Laboratory-based assessments took place in the Human Performance Laboratory at the University of Newcastle, Callaghan campus. Assessments occurred at baseline, 3, 6 and 18 months and an extra 9 months assessment for the control participants.

#### Anthropometry

**Height: **measured to 0.1 cm using the stretch stature method on a Harpenden portable stadiometer (Holtain Limited, Dyfed, Britain). Height was measured twice, with accepted values within 0.3 cm. A third measure was taken if measurements were outside the acceptable range. The average of the two acceptable measures will be reported.

**Weight: **measured in light clothing, without shoes on a digital scale to 0.01 kg (CH-150kp, A&D Mercury Pty Ltd, Australia). Weight was measured twice, with accepted values within 0.1 kg. A third measure was taken if measurements were outside the acceptable range. The average of the two acceptable measures will be reported.

**Waist circumference: **measured to 0.1 cm using a non-extensible steel tape (KDSF10-02, KDS Corporation, Osaka, Japan). Waist circumference was measured at two points (i) level with the umbilicus and (ii) at the narrowest point between the lower costal border and the umbilicus. Two measures were taken at each site, with accepted values within 0.5 cm. Further measures were taken if measurements were outside the acceptable range. The average of the two acceptable measures will be reported.

**Body mass index (BMI): **calculated from height and weight (kg/m^2^).

#### Dietary intake

Dietary intake is assessed using the Australian Eating Survey (AES). AES is a 120-item semi-quantitative FFQ, used previously in Australian youth up to 16 years [15] and currently being validated in both adult males and females. Portion sizes for individual food items were generated by the Australian Bureau of Statistics (ABS) [16] and unpublished data from the 1995 Australian National Nutrition Survey; or the "natural" serving size for common items such as a slice of bread. Subjects were asked about frequency of their consumption over the previous six months with frequency options ranging from 'Never' up to '4 or more times per day' but varying depending on the food item. Twenty-one questions related directly to the intake of vegetables and 11 questions related to fruit. Seasonal availability of some fruits will be considered in the nutrient analysis.

Nutrient intakes from the FFQ were computed from the most current food composition database of Australian foods available, the Australian AusNut 1999 database (All Foods) Revision 17 and AusFoods (Brands) Revision 5 (Australian Government Publishing Service, Canberra) to generate individual mean daily macro-and micro-nutrient intakes.

The AES includes questions about the total number of daily serves of fruit, vegetables, bread, dairy products, eggs, fat spreads, sweetened beverages and snack foods, as well as asking the type of bread, dairy products and fat spreads used. Twelve questions relate to food-related behaviours, including items on frequency of take-away food consumption and eating while watching television.

#### Eating behaviour

The Three Factor Eating Questionnaire-R18, (TFEQ-R18) is a self-assessment questionnaire to measure cognitive and behavioural components of eating in obese populations across three scales of cognitive restraint, uncontrolled eating and emotional eating [17]. The 18 items are coded on a four-point scale with higher values indicating the behaviour is expressed more frequently or the statement is more correct for the participant.

#### Physical activity

**International Physical Activity Questionnaire **(short form): data from this questionnaire was used to estimate total MET-minutes/week and to classify participants into either high, medium or low physical activity categories according to the IPAQ Scoring Protocol [18].

**Pedometers: **physical activity was objectively measured using pedometers (Yamax SW700; Yamax Corporation, Kumamoto City, Japan). At baseline, participants were instructed on how to attach the pedometers (at the waist on the right hand side) and asked to remove the pedometers only when sleeping, when the pedometer might get wet (e.g. swimming, showering) or during contact sports. Participants were asked to wear pedometers for seven consecutive days and keep to their normal routine. At the end of the day participants were instructed to record their steps on a pedometer record sheet and reset their pedometers to zero. Participants were instructed to record if they did an activity like cycling, swimming, contact sports or another activity that did not involve stepping and include details (type of activity and duration), or if they forgot to wear their pedometer for an amount of time. Participants will be included in all analyses if they completed at least four weekdays of pedometer monitoring. The average of existing days will be imputed for participants who have included at least four days of data.

#### Online diet and physical activity diary

The Australian Biggest Loser Club online food and exercise diary is powered by the database (International Food and Exercise Database). This database holds the information on over 70,000 foods including 20,000 Australian foods, 250,000 food portions, 5000 meals and recipes, and 1000 exercises. It is derived from a combination of methods including: 1) nutrition information from branded products collected by SP Health; 2) recipes to derive commonly eaten foods developed by an Accredited Practising Dietitian; and 3) sources such as the Australian food database (NUTTAB 2006, Canberra). The METs of exercises have been obtained predominantly from the Ainsworth [19,20] compendium of activities, and all exercise instructions have been compiled by SP Health.

#### Indicators of change in Health Status and Quality of Life

**Blood pressure and resting heart rate: **systolic blood pressure, diastolic blood pressure and heart rate were measured using an automated blood pressure monitor (NISSEI/DS-105E digital electronic blood pressure monitor (Nihon Seimitsu Sokki Co. Ltd., Gunma, Japan) under standardized conditions. Subjects were seated for five minutes before the first blood pressure measurement and a rest period of two minutes between measures. Blood pressure was measured three times. Further measurements were taken if the blood pressure or resting heart rate values fell outside of the acceptable ranges i.e. systolic within 10 mmHg, diastolic within 10 mmHg (preferably 5 mmHg) and resting heart rate within 5 bpm. The mean of the two closest systolic pressures and the diastolic pressure paired to them will be reported. The mean of the two lowest resting pulse pressures will be used.

**Blood sample biomarkers: **blood samples were collected after an overnight fast and analysed using standard automated techniques at a single National Association of Testing Authorities accredited pathology service. The samples were analysed for lipids (Total Cholesterol, Low Density Lipoprotein (LDL) and High Density Lipoprotein (HDL) cholesterol, and triglycerides), thyroid function and liver function, C-reactive protein, glucose and insulin. Additional samples have been stored for future analysis of additional biomarkers.

**Quality of Life: **SF-36, version 2.0 (QualityMetric Incorporated, Lincoln, RI, USA) is a multi-purpose, generic short-form health survey consisting of 36 questions using an 8-scale profile of functional health and well-being scores and psychometrically-based physical and mental health summary measures and scored according to standardised procedures [21]. It has been used widely in surveys of general populations and in differentiating the health benefits produced by a range of different treatments.

#### Use of Internet features and satisfaction with weight loss program

A process evaluation questionnaire was developed from a previous study [22] to assess the use of website features and satisfaction with the web based program, and completed by participants after 3, 6 and 18-months. Objective measurement of participants' use of the website features (e.g. log-ins to specific program features, diary entries, post to bulletin boards) was collected and stored by SP Health Pty Ltd throughout Phase 1 and Phase 2.

#### Timelines for Programs

Baseline assessments occurred in one session and participants in the two intervention groups were able to log on and commence the programs immediately after this initial assessment. This session was conducted early in the morning and participants were offered breakfast following the blood collection, anthropometric and blood pressure assessments. Participants repeated these assessments at 3, 6, and 18 months with the control group having their post-intervention assessments at 9 and 21 months (due to their 3 months wait-list period). To maximize adherence to these sessions, participants were asked to book into an assessment time that suits them using the online scheduling system, Doodle http://doodle.com or alternatively via a telephone call. In addition, for participants difficult to contact other methods were employed such as text messaging and mailing letters. A contribution towards parking and travel costs of $AU10 was given to those attending assessment sessions.

### Data analysis

Analysis will be on an intention-to-treat basis. Analysis of covariance will be used to test for differences in weight loss between groups at 3 months. The model will be fitted using linear regression with weight at 3 months as the outcome variable, treatment group as the predictor variable of interest and weight at baseline included as a covariate. Other independent variables included in the model will be: gender, kilojoule intake at baseline and physical activity at baseline. Differences in weight loss between groups in the weight maintenance phase will be analysed using generalised linear mixed models (GLMM). The outcome in the model will be the individual's weight at the post treatment assessments, the main predictor of interest will be treatment group and baseline weight will be included as a covariate. Other independent variables included in the model will be: time, gender, kilojoule intake at baseline and physical activity at baseline.

Statistical significance of the primary efficacy analysis (at three months and 18 months) will be based on Hochberg's multiple testing procedure with the family wise error rate for each time point held at 2.5%. All secondary hypothesis tests will be performed using a 2-sided 5% significance level. In addition, linear regression and GLMM will be used to describe relationships among the various dependent and independent variables.

## Discussion

The study described in this paper is one of the first randomized controlled trials of its kind in Australia. The results of this study of a commercially available program will provide much needed information about the efficacy of treatment approaches using the internet in real world settings. It is important internationally because web-based weight management interventions for adults to date have only shown moderate success and commercially available online programs have not been tested for efficacy using a robust study design [13].

This RCT addresses some of the short-falls in current web-based weight management interventions by (i) incorporating a large sample size compromised of representative samples of both men and women, and an intention to treat analysis plan; (ii) having an 18-month follow-up period, thereby allowing assessment of medium term program effectiveness; (iii) including several important secondary outcomes (metabolic profile, self-monitoring of dietary intake and physical activity, quality of life), and (iv) inclusion of a maintenance of lost weight phase. Data demonstrating whether the study yields positive long-term results from the end of the maintenance phase or not will allow other research groups to benefit from our experience and facilitate the implementation of well-designed RCTs to address the lack of quality web-based interventions. Importantly it will also ascertain whether commercial providers can deliver efficacious web-based programs targeting this important public health issue.

## Funding

Australian Research Council Linkage Project grant (2009-2012) (#LP0990414, G0189752), with SP Health as the Industry Partner Organisation (#G0189753). CEC is supported by a National Health and Medical Research Council Australian Career Development Award Research Fellowship (#6315005). K Fletcher is supported by an ARC APAI scholarship. M Neve is supported by an Australian Postgraduate Award (APA).

## Competing interests

CEC is a dietetic consultant to SP Health Co. M Neve received a PhD scholarship supplement from SP Health Co, and P Jones is employed as an Accredited Practising Dietitian by SP health Co. All other authors declare that they have no competing interests.

## Authors' contributions

CC, PM, RC were responsible for the design of study and this paper. PMcE provided advice on sample size, randomization and statistical analysis. CC, PJ, PM, RC and Anna Crook were responsible for the development of the enhanced intervention and the maintenance of lost weight program. All authors were responsible for drafting and revising the manuscript and have approved the final version.

## Pre-publication history

The pre-publication history for this paper can be accessed here:

http://www.biomedcentral.com/1471-2458/10/669/prepub
